# Plant-Based Colloidal Delivery Systems for Bioactives

**DOI:** 10.3390/molecules26226895

**Published:** 2021-11-16

**Authors:** Yunbing Tan, David Julian McClements

**Affiliations:** 1 Department of Food Science, University of Massachusetts Amherst, Amherst, MA 01003, USA; ytan@umass.edu; 2 Department of Food Science & Bioengineering, Zhejiang Gongshang University, 18 Xuezheng Street, Hangzhou 310018, China

**Keywords:** bioactives, plant-based delivery, colloidal systems, functionality, emulsions

## Abstract

The supplementation of plant-based foods and beverages with bioactive agents may be an important strategy for increasing human healthiness. Numerous kinds of colloidal delivery systems have been developed to encapsulate bioactives with the goal of improving their water dispersibility, chemical stability, and bioavailability. In this review, we focus on colloidal delivery systems assembled entirely from plant-based ingredients, such as lipids, proteins, polysaccharides, phospholipids, and surfactants isolated from botanical sources. In particular, the utilization of these ingredients to create plant-based nanoemulsions, nanoliposomes, nanoparticles, and microgels is covered. The utilization of these delivery systems to encapsulate, protect, and release various kinds of bioactives is highlighted, including oil-soluble vitamins (like vitamin D), ω-3 oils, carotenoids (vitamin A precursors), curcuminoids, and polyphenols. The functionality of these delivery systems can be tailored to specific applications by careful selection of ingredients and processing operations, as this enables the composition, size, shape, internal structure, surface chemistry, and electrical characteristics of the colloidal particles to be controlled. The plant-based delivery systems discussed in this article may be useful for introducing active ingredients into the next generation of plant-based foods, meat, seafood, milk, and egg analogs. Nevertheless, there is still a need to systematically compare the functional performance of different delivery systems for specific applications to establish the most appropriate one. In addition, there is a need to test their efficacy at delivering bioavailable forms of bioactives using in vivo studies.

## 1. Introduction

Recently, many sectors of the food industry are focusing on the creation of high-quality plant-based foods that are designed to accurately mimic the sensory attributes of animal-based foods, such as meat, seafood, eggs, and milk [1]. These plant-based foods are being created in response to consumer demand for products that are better for the environment, human health, and animal welfare. Indeed, this is one of the fastest-growing segments of the modern food industry, with rapid advances being made in all food categories [2]. Plant-based foods do not have the same nutritional profiles as the animal-based ones they are designed to replace. They are often deficient in some key nutrients, such as vitamin B_12_, vitamin D, omega-3 fatty acids, iron, and zinc. Consequently, there is interest in fortifying them with these nutrients. In addition, the healthiness of these products may be further enhanced by fortifying them with nutraceuticals, such as carotenoids, curcuminoids, and polyphenols. In this article, we focus on the fortification of plant-based foods with oil-soluble bioactive agents since these are difficult to incorporate into many food and beverage products and often have a low bioavailability after consumption. In particular, we focus on the utilization of nanoenabled delivery systems that are entirely assembled from plant-based ingredients to improve the functional performance of these hydrophobic bioactives.

## 2. Plant-Based Components

An overview of the different kinds of plant-based ingredients that can be used to assemble nanoenabled delivery systems is given in this section.

### 2.1. Lipids

Lipids can be derived from a variety of oil-rich plants from around the globe, including seeds, pulps, and fruits. Oilseeds such as those obtained from soybeans, sunflowers, peanuts, and corn are some of the most important commodities used for this purpose because of their high lipid contents and ease of extractability. In terms of fatty acid composition, most plant oils comprise a high percentage of unsaturated fatty acids [3]. Consumption of unsaturated fatty acids has been linked to various health benefits, including reducing the risk of cardiovascular disease and inflammation [4]. In particular, omega-3 fatty acids, such as those found in flaxseed oil and algal oil, have been demonstrated to be beneficial to human health (provided they are not oxidized). In addition, plant oils are an important source of oil-soluble vitamins and nutraceuticals, such as vitamin D, phytosterols, and carotenoids [5]. Plant oils have a relatively low melting point due to their high unsaturated fatty acid content, and so they tend to be liquid at ambient temperatures. Nevertheless, there are some exceptions, such as coconut oil, cocoa butter, and palm oil. The fluidity of many plant-based oils at room and refrigerator temperatures makes them suitable for forming nanoemulsion-based delivery systems. The solidity of some plant-based oils under these conditions means they can be used to create solid lipid nanoparticles (SLNs) or nanostructured lipid carriers (NLCs) [6].

Fluid oils can be changed into solid fats using hydrogenation to convert the unsaturated fatty acids into saturated ones, thereby increasing their melting point [7]. However, this method is being used less frequently because of the potential formation of trans fatty acids as a side product. In addition, plants generate different types of waxes that are also solid under ambient conditions. These waxes have been used to form oleogels or emulsified oleogels that can be used as plant-based delivery systems in foods [8,9,10].

Plants also contain phospholipids, such as those found in soy or sunflower lecithin ingredients, which can be considered polar lipids. Phospholipids contain both hydrophilic and lipophilic components, and so they can be used as emulsifiers to form and stabilize the oil droplets of fat particles in nanoemulsions, SLNs, or NLCs. Furthermore, chemical or enzymatic methods can be used to increase the hydrophilicity of phospholipids by removing a fatty acid chain to produce lysolecithin, which can improve their emulsifying ability [11]. Phospholipids are also widely used to form nanoliposomes, which are colloidal particles assembled from one or more phospholipid bilayers [12]. Other lipophilic materials can also be used to form plant-based nanoparticles. For instance, quillaja saponin is a surface-active substance belonging to the terpenoid family that can be isolated from the bark of a tree. Previous studies have shown that quillaja saponin can be used to form small oil droplets or fat particles that are stable over a broad range of environmental conditions (such as temperature, pH, and salt concentration) [13]. It can, therefore, also be used to formulate nanoemulsions, SLNs, or NLCs.

### 2.2. Proteins

Several kinds of plant proteins can be used to assemble plant-based colloidal delivery systems, including those from soybeans, peas, rice, and wheat. In particular, soy proteins have been widely studied for decades for this purpose because of their good emulsifying, gelling, and foaming abilities [14]. In addition to these well-known plant proteins, research is being carried out to discover novel sources of plant proteins and also to test their potential application, such as those from pumpkin seed [15], longan pulp [16], cowpeas [17], tomato seeds [18], and walnut protein [19]. Many of these plant proteins are extracted as a byproduct from other processes in the food industry (such as oil or starch refining), and therefore their application as functional ingredients would greatly increase the value of the food chain.

Like many animal proteins, many plant proteins are amphiphilic globular molecules that can be used as emulsifiers because of their good surface activity and film-forming properties. However, the functionality of plant proteins depends on their molecular characteristics, which depend on their botanical origin, isolation procedure, and processing steps. In particular, the molecular weight, amino acid sequence, conformation, surface hydrophobicity, surface charge distribution, and flexibility of these proteins influence their solubility, emulsifying, gelling, foaming, and water-holding properties, as well as their digestibility [20]. After extraction, many plant proteins exhibit limited functionality. For this reason, various approaches have been utilized to modify the structure and functional performance of plant proteins. For instance, they can be covalently or non-covalently attached to polysaccharides, polyphenols, or other components to modulate their properties [21]. Moderate hydrolysis of plant proteins can greatly improve their solubility and emulsifying properties [15]. Controlled aggregation of proteins can be used to create colloidal particles that are suitable for forming and stabilizing Pickering emulsions [20]. Mixtures of proteins have also been explored as a promising approach to improve their functional performance and nutritional value [20].

### 2.3. Polysaccharides

Polysaccharides, such as starch and non-starch polymers, are a major component of many plants that can be used as functional ingredients in foods. In addition, some polysaccharides can be extracted from microbial fermentation or from seaweeds, such as microalgae and algae. Numerous types of polysaccharide-based ingredients are commercially available for application in foods, including starch, cellulose, alginate, carrageenan, pectin, agar, gellan gum, xanthan gum, gum arabic, and their derivatives. Many of these ingredients can be used to form plant-based colloidal particles. Polysaccharides are natural polymers that can vary considerably in their molecular weights, from several kilodaltons to several thousand kilodaltons. The structure of polysaccharide molecules depends on the type, number, sequency, and bonding of the monomers they contain. However, polysaccharides may also be present in complex structural organizations within plants. The molecular and functional properties of polysaccharides often depend on the extraction conditions applied [22]. Polysaccharides vary considerably in their solubility, thickening, gelling, foaming, emulsifying, and water-holding properties, as well as the way they behave in complex food matrices. Consequently, it is important to select polysaccharide ingredients that exhibit the functional attributes required to create a colloidal delivery system appropriate for a specific application [23].

Polysaccharides vary widely in their physiological effects, which should be accounted for when designing delivery systems for plant-based foods. Some starches are rapidly digested in the mouth and small intestine, leading to spikes in blood glucose levels, whereas dietary fibers are not digested in the upper gastrointestinal tract, which can have beneficial effects. First, they may promote satiety, thereby reducing calorie consumption. Second, the addition of polysaccharides can delay the digestion process of many micronutrients [24], for instance, lipids and carbohydrates, thereby reducing their tendency to cause spikes in the bloodstream [25,26]. Third, some dietary fibers are fermented in the colon, which can modulate the composition of the gut microbiota leading to health benefits [27]. Fourth, some polysaccharides exhibit antioxidant, antimicrobial, anti-inflammatory, and anticancer activities, which can also enhance human health [28].

### 2.4. Other Plant-Based Ingredients

In addition to the plant-based macronutrients discussed above, there are various micronutrients and nutraceuticals (phytochemicals) that also can be extracted from plants and used as functional ingredients in colloidal delivery systems. For instance, carotenoids are natural pigments found in many brightly colored fruits and vegetables that are precursors of vitamin A and exhibit various other health benefits [29,30]. Plants also contain various kinds of phenolic substances that exhibit health effects, including curcuminoids, anthocyanins, catechins, and tannins [31,32], as well as other bioactive substances like tocopherols, tocotrienols, coenzyme Q10, and bioactive peptides [33]. Fortification of plant-based foods with these components may therefore lead to important health benefits. 

## 3. Ingredient Functionality

### 3.1. Emulsifying

**Figure 1:**
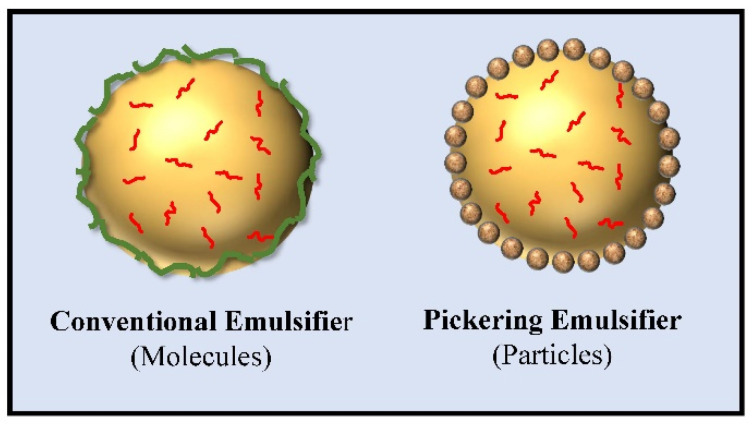
Emulsion stabilization mechanisms: molecular versus particle emulsifiers.

Many plant ingredients are amphiphilic molecules that can adsorb to oil-water interfaces and stabilize oil droplets or fat particles, especially proteins, phospholipids, and saponins (Figure 1). These substances can therefore be used to form nanoemulsions, SLNs, or NLCs. Typically, proteins and saponins (which are predominantly hydrophilic) are dissolved in the water phase prior to homogenization, whereas phospholipids (which have balanced hydrophilic/hydrophobic characters) may be dispersed in the oil or water phases. The oil and water phases are then blended together and/or homogenized, which leads to the generation of small oil droplets. The amphiphilic molecules then adsorb to the oil-water interface and prevent the droplets from aggregating by generating steric and electrostatic repulsive forces (when the pH is far from the isoelectric point) [20]. For instance, molecular forms of plant proteins (like soy) have been shown to be effective emulsifiers for forming and stabilizing oil-in-water emulsions [14]. In addition, particulate forms of plant proteins can be used to form and stabilize emulsions through a Pickering mechanism (Figure 1) [20]. For example, corn zein, which is a strongly hydrophobic protein, has been widely investigated as a Pickering stabilizer in emulsions [34,35]. In addition, protein nanoparticles formed by controlled thermal denaturation of soy protein have also been used as Pickering stabilizers [36]. There is little information about the emulsifying properties of many other hydrophobic cereal proteins, such as those isolated from wheat and oat, which might also be good Pickering stabilizers.

In addition to plant proteins, several plant-derived phospholipids and polysaccharides can also be used as emulsifiers, such as soy lecithin, gum arabic, and modified starch [37]. However, compared to proteins, these substances are usually not as effective at forming fine oil droplets during homogenization, with the average diameter of the oil droplets formed often being over a few to tens of micrometer [37,38]. Quillaja saponin is another promising non-protein emulsifier, which can be considered to be an anionic small-molecule surfactant. This surfactant has been shown to generate oil droplets less than 200 nm in diameter that remain stable to aggregation over a wide range of environmental conditions [39].

### 3.2. Gelling

Many plant proteins and polysaccharides have good gelling properties. Under appropriate conditions (such as concentration, temperature, pH, ionic composition, and/or enzyme activity), they may crosslink with their neighbors through non-covalent bonds (hydrogen bond, electrostatic forces, Van der Waals forces, and hydrophobic interactions) or covalent bonds, thereby leading to a sol-gel transition. Protein gels have been prepared using soy, pea, and wheat proteins through both heat-set and cold-set methods. In the heat-set method, a sufficiently high concentration of protein is dissolved in a solution with a controlled pH and ionic composition. The solution is then heated above the thermal denaturation temperature of the proteins, which causes them to unfold and form hydrophobic and disulfide crosslinks with each other. Alternatively, a cold-set gelation method can be used. In this case, the proteins are heated above their thermal denaturation temperature under solution conditions that cause them to unfold but not form a 3D network. This can be achieved by controlling the pH and ionic strength, so there is an electrostatic repulsion between the unfolded proteins. The solution can then be cooled to room temperature and gelled by altering the pH or ionic composition to reduce the electrostatic repulsion between the protein molecules [40]. Other commonly used gelling mechanisms include enzymatic crosslinking using transglutaminase or laccase, as well as acidification crosslinking using glucono-δ-lactone or microbial fermentation [41]. The textual properties of the final gel depend on protein type, protein concentration, gelation mechanism, gelling conditions, and various other factors. For instance, rubisco was shown to form strong gels at relatively low concentrations [42]. Typically, the gel strength increases with increasing protein concentration [43], which may be important for applications where robust microgels are required.

Many food-grade polysaccharides are also able to form gels, with the gelling mechanism depending on polysaccharide type. For instance, some polysaccharides require salt (e.g., alginate and calcium), some require cooling (e.g., agar), some require heating (e.g., methylcellulose), and some require specific pH and osmotic conditions (e.g., high methoxy pectin requires acids and sugars to induce gelation). The gels prepared from different polysaccharides or through different mechanisms have different microstructures, which impacts their appearance (clear or opaque), textures (hard or soft; brittle or rubbery), and water holding properties (good or bad). Therefore, an appropriate polysaccharide should be selected as a gelling agent depending on the requirements of the application. Often, a low polysaccharide concentration is sufficient to induce gelation [44].

Gels prepared from single components sometimes do not have the functional attributes required for a specific application. In this case, gels with novel or improved properties can be created using mixtures of different components, such as protein-protein, polysaccharide-polysaccharide, or protein-polysaccharide mixtures [45].

### 3.3. Structure Forming

Plant-based ingredients can be used to form specific structures within food products, which give desirable physicochemical or sensory attributes. For instance, phase separation, stirring, and gelling of protein-polysaccharide mixtures can lead to fiber-like structures and textures being formed that somewhat resemble those found in meat and seafood [1]. Alternatively, layer-by-layer electrostatic deposition can be formed to form multilayer coatings around particles. In this case, the polymers used should have opposite electrical charges so that anionic groups on one polymer bind to cationic groups on another polymer.

## 4. Delivery System Types

**Figure 2:**
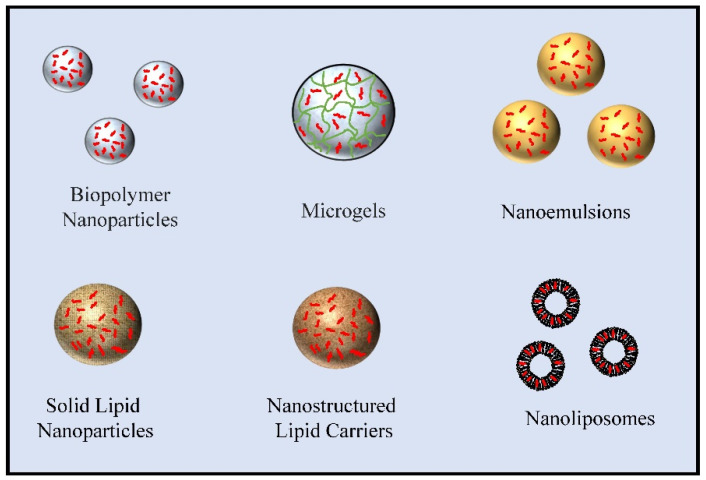
Schematic diagram of different types of delivery systems that can be assembled from plant-based ingredients.

In this section, we highlight different kinds of colloidal delivery systems that can be assembled entirely from plant-based ingredients (Figure 2). These delivery systems can be used to encapsulate, protect, and release various types of hydrophobic bioactive agents, including oil-soluble vitamins, omega-3 oils, and nutraceuticals.

### 4.1. Biopolymer Nanoparticles

Researchers have investigated the suitability of biopolymer nanoparticles for encapsulating hydrophobic bioactive substances [46]. The main advantages of these systems are they can be assembled from proteins and/or polysaccharides and can be designed to have good physicochemical stability and bioavailability. Biopolymer nanoparticles have been developed for the encapsulation of different types of hydrophobic vitamins and nutraceuticals, including vitamin D [47], vitamin E, lutein [48], resveratrol [49], and curcumin [50]. The hydrophobic bioactive agents are usually trapped within hydrophobic domains inside the nanoparticles. The composition and structure of biopolymer nanoparticles, which depend on the nature of the ingredients and fabrication methods used to manufacture them, influence their retention/release properties, physicochemical stability, and gastrointestinal fate. For instance, researchers have compared the performance of protein-based nanoparticles with phospholipid-based nanoliposomes for encapsulating curcumin [51]. The curcumin encapsulated within the protein (zein) nanoparticles was more stable than that encapsulated within the phospholipid nanoparticles, which was attributed to the antioxidant activity of the proteins. There is ongoing research to identify new plant-based materials to prepare biopolymer nanoparticles, such as lignin [52]. In addition, researchers are examining combinations of different biopolymers to obtain improved properties or novel functionalities in the nanoparticles. For instance, coating zein nanoparticles with carboxymethyl chitosan improved the encapsulation efficiency, photochemical stability, and gastrointestinal release of vitamin D [47]. The addition of tea saponins has been shown to improve the encapsulation efficiency, photostability, and thermal stability of resveratrol in protein nanoparticles [49]. Controlling the electrostatic interactions in aqueous solutions by altering the pH or ionic strength can also be used to modify the functional performance of nanoparticles. For instance, calcium addition to zein-propylene glycol alginate-tea saponin complexes led to aggregation and the formation of a more compact structure, which retarded the release of resveratrol under simulated gastrointestinal conditions [49].

Many different methods are available to prepare biopolymer nanoparticles. Antisolvent precipitation is one of the most widely used. In this method, hydrophobic biopolymers (such as zein or gliadin) and nutraceuticals (such as curcumin) are first dissolved in a concentrated aqueous organic solvent (such as 80% ethanol and 20% water). This solution can then be slowly added to water, which causes the hydrophobic proteins to precipitate with each other, leading to the formation of nutraceutical-loaded protein nanoparticles [51]. The organic solvent should then be removed, which can be achieved by heating the system under a vacuum. However, it is possible that some organic solvent remains in the final sample.

### 4.2. Microgels

Microgel systems are also prepared from biopolymer molecules, but they tend to be bigger and have a more open gel network structure inside them that contains a relatively large amount of water. In contrast, biopolymer nanoparticles tend to be relatively small and dense and contain much less water. There are many methods available to prepare microgels [53], but the most used ones are the injection-gelation and coacervation methods. Typically, the hydrophobic vitamins or nutraceuticals are first trapped inside lipid nanoparticles (such as oil droplets, SLNs, NLCs, or nanoliposomes), which are then trapped inside the microgels using a method that depends on the fabrication technique employed. For instance, for the injection-gelation method, the bioactive-loaded lipid nanoparticles are mixed with a biopolymer solution, which is then injected into a gelling solution. This leads to the formation of microgels containing bioactive-loaded lipid nanoparticles. In the coacervation method, the bioactive-loaded lipid nanoparticles are mixed with a solution containing two polymers, which are then made to interact with each other and form biopolymer-rich microgels with the bioactive inside. This is often achieved by mixing a bioactive-loaded nanoemulsion with a solution that contains a protein and anionic polysaccharide. Under appropriate pH conditions (around the isoelectric point), the anionic groups on the polysaccharide bind to cationic groups on the protein surfaces, which leads to coacervate formation. 

The composition and structure of the hydrogel network surrounding the lipid nanoparticles can be designed to protect the bioactives from chemical degradation or to control the release of the bioactive agents (such as extended or triggered release). For example, encapsulation of omega-3 oil droplets within calcium alginate/caseinate microgels formed using the injection-gelation method has been shown to significantly inhibit lipid oxidation, as seen by a reduction in primary (hydroperoxides) and secondary (TBARS) reaction products [54]. Other researchers have shown that encapsulation of β-carotene within calcium alginate microgels improved its stability to storage and heating [55]. The efficacy of microgels at enhancing the storage stability of encapsulated substances also depends on environmental conditions. For instance, the encapsulation of curcumin in chitosan microgels significantly increased its stability under neutral pH conditions, but this protective effect was much less under acidic conditions [56]. In general, the chemical stability of encapsulated substances can be improved by various mechanisms, including: (i) the inclusion of antioxidant components (such as some proteins and polyphenols) inside the microgels; (ii) the inclusion of components that can chelate pro-oxidant transition metals (such as some proteins and polysaccharides) inside or outside the microgels; (iii) the formation of a 3D biopolymer network inside that microgels that acts as a physical barrier that keeps prooxidants away from the encapsulated substances.

**Figure 3:**
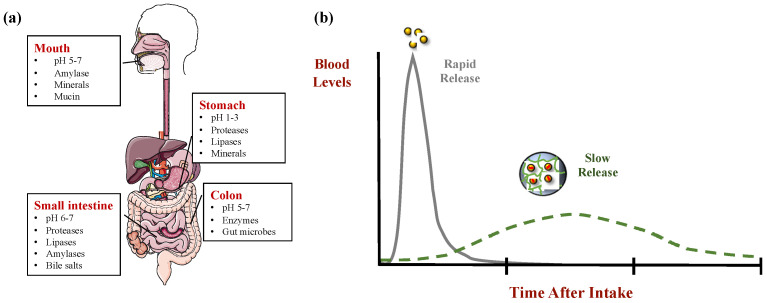
(**a**) Illustration of the environmental conditions in the gastrointestinal tract; (**b**) Rapid- and slow-release patterns of encapsulated bioactives.

Microgel beads have also been used to inhibit the digestion of encapsulated lipids by retarding the movement of lipase molecules through the biopolymer network and to the oil droplet surfaces (Figure 3). As an example, encapsulation of lipid droplets within alginate beads significantly reduced the rate and extent of lipid digestion under simulated gastrointestinal conditions [57,58]. The lipid digestion profiles could be modulated by altering the external dimensions and internal pore size of the microgels. For instance, lipid digestion is slower for microgels with smaller internal pores because this inhibits the diffusion of lipase through the biopolymer network [59,60]. In addition, it is slower for larger microgels than smaller ones because the lipase molecules have further to diffuse to reach the encapsulated lipid droplets [60,61]. However, these protective microgels must be designed to retain their physical integrity under the environmental conditions under which they are used; otherwise, they will just release the encapsulated substances.

Microgels can also be designed to control the release profiles of encapsulated substances, such as burst, prolonged, or triggered release. This can be achieved through a variety of mechanisms, including diffusion, swelling, erosion, and disintegration [62]. In diffusion, some of the bioactive molecules simply diffuse through the interior of the microgels and are then released. The rate of release can be extended by increasing the external dimensions (diameter) of the microgels or by decreasing the internal pore size of the biopolymer network. In swelling, the microgel is designed to expand under certain conditions, which leads to an increase in the pore size. As a result, small bioactive-loaded lipid nanoparticles can then leak out of the microgel. Typically, swelling occurs when there is an increase in the repulsion between the biopolymer molecules or an alteration in their conformation in response to an environmental change, such as pH, ionic strength, or temperature. For example, electrically charged polymers may swell when the ionic strength decreases because of the increased repulsion between them. Erosion or disintegration occurs when the microgels are physically or chemically disrupted, thereby releasing the encapsulated substances. For erosion, this starts from the exterior of the microgels and works inwards, but for disintegration, this occurs throughout the entire volume of the system. These processes may occur because of weakening of the attractive forces between the biopolymer molecules (e.g., a reduction in electrostatic attraction between oppositely charged biopolymers at high salt levels) or due to fragmentation of the biopolymer molecules (e.g., the hydrolysis of starch or protein molecules in the presence of amylase or protease). These phenomena can be utilized to develop microgels that will release their encapsulated components under specific conditions.

### 4.3. Nanoemulsions

Oil-in-water nanoemulsions have been widely used as delivery systems for hydrophobic vitamins and nutraceuticals [63]. These are thermodynamically unstable systems consisting of emulsifier-coated oil droplets dispersed in water. They are like conventional emulsions, except they contain smaller oil droplets (diameter < 200 nm). The main advantage of nanoemulsions as delivery systems is that they can easily be prepared from plant-derived ingredients using common food processing operations like mixing and homogenization [6]. In addition, the small droplet size often leads to improved physical stability and enhanced bioavailability.

Nanoemulsions can be formed using either high- or low-energy methods, but the former is the most commonly employed [63]. High-energy methods use mechanical devices capable of generating intense, disruptive forces to break up the oil and water phases into fine droplets. The most used are high shear mixers, colloid mills, high-pressure valve homogenizers, microfluidizers, and sonicators. Low-energy methods rely on the spontaneous generation of nanoemulsions when a solution or environmental conditions are changed, such as composition, temperature, or salt concentration. However, these methods are typically not suitable for forming plant-based nanoemulsions. The selection of an appropriate homogenization method depends on the nature of the ingredients used, the type of end-product being produced, and the functional attributes required.

Several researchers have examined the suitability of nanoemulsions for improving the functionality of hydrophobic bioactive agents. The encapsulation of curcumin in a nanoemulsion was shown to increase its chemical stability during storage, which was attributed to the fact that curcumin degrades more rapidly when exposed to aqueous environments [56]. As a result, the rate of curcumin degradation increased as the lipid droplet size decreased because then the specific surface area of the oil droplets increased, thereby exposing more curcumin to the surrounding aqueous phase. Other studies have shown that encapsulation of curcumin within nanoemulsions increases its water dispersibility, physical stability, and bioavailability [64]. Several studies have compared the performance of nanoemulsions with other delivery systems. For instance, nanoemulsions were more effective at improving the bioaccessibility of curcumin than biopolymer nanoparticles, but their loading capacity was much lower [51].

**Figure 4:**
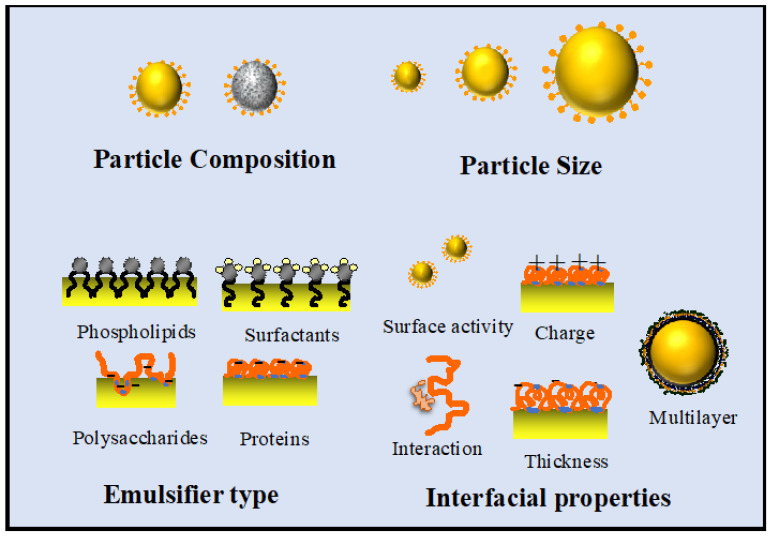
Some critical factors that determine the functional performance of nanoemulsion delivery systems.

The functional performance of nanoemulsions as delivery systems depends on numerous factors, including the oil phase composition, droplet size, emulsifier type, interfacial properties, and additives (Figure 4). The interfacial layer formed by the emulsifier is a critical component of nanoemulsion delivery systems. First, the emulsifier should reduce the interfacial tension between the oil and water phases, which promotes the creation of small oil droplets during homogenization. Second, the interfacial layer should generate strong repulsive forces that inhibit droplet aggregation, which are usually steric and/or electrostatic in origin. Third, the interfacial layer is the site for many chemical and enzymatic reactions, such as oxidation and digestion, and so its composition should be manipulated to control these reactions [65]. Many researchers have examined the impact of plant-derived emulsifiers on the performance of nanoemulsions. Some plant proteins can be used as molecular emulsifiers to form and stabilize emulsions, such as pea, legume, or fava bean proteins [66]. However, these nanoemulsions are prone to aggregation under certain conditions, such as high salt contents, pH values near the isoelectric point, and temperatures above the thermal denaturation temperature. Several strategies have been deployed to overcome these problems. Plant proteins can be chemically or enzymatically modified to improve their emulsifying properties, e.g., using deamidation or hydrolysis [67]. In addition, proteins can be covalently linked to other molecules (such as polysaccharides or polyphenols) to form conjugates with improved functional performance. For instance, protein-polysaccharide conjugates formed through the Maillard reaction (or other reactions) typically have better solubilities and emulsifying properties than proteins alone [68]. Alternatively, protein-polysaccharide complexes, which are held together by physical interactions (usually electrostatic attraction), can also be used to improve the functional performance of proteins [69]. Many studies have shown that the formation of interfacial protein-polysaccharide complexes improves the resistance of oil droplets to environmental changes [70,71]. More complicated conjugates or complexes can be formed with enhanced functional properties by linking three or more components together, such as a protein, polysaccharide, and polyphenol. These ternary complexes can form and stabilize nanoemulsions like regular emulsifiers, but they also provide antioxidant properties because of the polyphenols that can retard oxidation reactions [72,73].

The gastrointestinal fate of nanoemulsions can also be controlled by manipulating the interfacial layers. For instance, the interfacial layer can be designed to inhibit the attachment of bile salts or lipase to the oil droplet surfaces, which can be achieved by using strongly surface-active emulsifiers that are difficult to displace [74] or by forming a mechanically robust coating that is difficult to disrupt [75]. In addition, the emulsifier might form droplets that are unstable to aggregation under gastrointestinal conditions, which leads to flocculation or coalescence, thereby reducing the surface area of the lipase to adsorb and inhibiting lipid digestion [76]. The properties of the interfacial layer can also be manipulated by controlling the aggregation state of the proteins. For instance, some plant proteins (such as soy protein) can form a protective gel layer around oil droplets by controlled thermal treatment and/or salt addition [14,77].

Oil phase composition is another critical factor affecting the design of nanoemulsion-based delivery systems. Using relatively polar oils, like many flavor or essential oils, leads to Oswald ripening, which significantly reduces nanoemulsion stability [78]. This instability mechanism manifests itself as an increase in droplet size over time due to the diffusion of oil molecules from small to large droplets. It can be inhibited by including a strongly hydrophobic oil (such as a long-chain triglyceride oil) into the oil phase prior to homogenization. The type of oil used also impacts the nutritional profile and chemical stability of nanoemulsions. For instance, oils containing high levels of polyunsaturated omega-3 fatty acids, such as flaxseed or algal oils, have good health benefits but are highly susceptible to oxidation. In contrast, oils containing high levels of saturated fatty acids, like coconut oil or medium-chain triglycerides, may have adverse health effects but are highly stable to oxidation.

Oil type also has a strong influence on the release and solubilization of hydrophobic vitamins and nutraceuticals under gastrointestinal conditions [79]. Typically, the digestion of long-chain triglycerides is slower than that of short or medium-chain ones because long-chain fatty acids are less soluble in gastrointestinal fluids, and so they may accumulate at the droplet surfaces, which inhibits the ability of the lipase to reach the ester bonds. Conversely, long-chain fatty acids form larger mixed micelles with a higher solubilization capacity for vitamins and nutraceuticals [80,81,82,83]. The hydrophobic interior of the mixed micelles formed should be large enough to accommodate the hydrophobic bioactive molecules; otherwise, the bioactives will precipitate, and the bioaccessibility will be low. The saturation degree of the fatty acids in the oil phase also influences the lipid digestion and solubilization processes and should therefore be optimized.

Various kinds of additives can be incorporated into nanoemulsions to improve their performance, including antioxidants to inhibit chemical degradation, weighting agents to retard creaming, ripening inhibitors to prevent Ostwald ripening, thickening, or gelling agents to modify texture and stability, and flavors and colors to enhance the sensory attributes.

### 4.4. Solid Lipid Nanoparticles and Nanostructured Lipid Carriers

Solid lipid nanoparticles (SLNs) and nanostructured lipid carriers (NLCs) are similar to nanoemulsions, but the oil phase is fully or partially solidified, respectively [6]. Thus, they typically consist of emulsifier-coated fat particles suspended in water. They can be produced using the same methods as nanoemulsions, but typically homogenization is carried out at a temperature above the melting point of the lipid phase, and then the system is cooled to promote crystal formation. SLNs and NLCs can be produced from plant-based lipids that are crystalline at ambient temperatures, such as coconut oil, cocoa butter, palm oil, hydrogenated oils, or some waxes. The crystalline nature of the lipid phase in SLNs or NLCs can improve the chemical stability of encapsulated hydrophobic bioactives by inhibiting their interaction with prooxidants in their environment. Moreover, it can also be designed to control the retention and release of encapsulated substances. However, SLNs and NLCs must be carefully formulated to avoid nanoparticle aggregation and bioactive expulsion when the lipid phase crystallizes [84,85]. The compact structure of the crystalline lipid phase in SLNs can also reduce their loading capacity for bioactive substances [86]. In addition, the expulsion of bioactive substances to the surfaces of lipid nanoparticles when the lipid phase crystallizes can increase their susceptibility to chemical degradation [87]. These problems can often be overcome by using NLCs rather than SLNs, since the solid structure inside these nanoparticles is less perfect, which prevents large changes in particle shape or expulsion of bioactives [88].

The physical state of the lipid nanoparticles influences lipid digestion and bioactive bioavailability. Lipid digestion is typically slower for solid fats than liquid ones since it is more difficult for lipase to hydrolyze crystalline fats [89]. A reduction in lipid digestion usually leads to a reduction in the release of the encapsulated bioactive molecules from the lipid nanoparticles. Consequently, SLNs or NLCs can be used to prolong the release of bioactives within the gastrointestinal tract [87]. It should be noted that solid lipid nanoparticles are still digested within the small intestine (albeit at a slower rate), and so they can still be used as delivery systems for hydrophobic bioactives [90]. For instance, β-carotene encapsulated in SLNs prepared from cocoa butter was shown to have a good bioaccessibility after lipid digestion under simulated small intestine conditions [87].

### 4.5. Nanoliposomes

Nanoliposomes are colloidal particles consisting of single or multiple concentric phospholipid bilayers [70,71,72,73,74,75]. They are like conventional liposomes but have smaller diameters (*d* < 200 nm). The bilayers form spontaneously due to the hydrophobic effect, but nanoliposomes are only metastable systems because their structures may change during storage. Nanoliposomes are often utilized to trap hydrophobic bioactives, which are integrated into the non-polar regions of the phospholipid bilayers (tail groups). They can also be used to encapsulate hydrophilic bioactives in their aqueous cores or in the polar regions of the phospholipid bilayers (head groups). Nanoliposomes may be formed from a single bilayer or from multiple bilayers arranged concentrically. Several methods are available for forming nanoliposomes, such as microfluidization, solvent deposition/evaporation, and antisolvent injection methods, but microfluidization is probably the most suitable approach for large-scale commercial production [63].

Nanoliposomes can be fabricated entirely from plant-based phospholipids, such as those found in soybean or sunflower lecithin. Consequently, they can be utilized as delivery systems in plant-based food applications. Nanoliposomes can be designed to be optically clear, which is advantageous for some applications. However, they do have some important disadvantages too. They are often expensive to prepare, they have a low loading capacity, and they are often unstable in complex food matrices.

## 5. Applications

In this section, we highlight the application of several plant-based colloidal delivery systems for improving the functional performance of hydrophobic bioactives (Table 1).

### 5.1. Oil-Soluble Vitamins

Oil-soluble vitamins (A, D, E, K) are hydrophobic micronutrients essential for human health, e.g., vitamin A is important for eye and skin health, vitamin D is important for bone health, and vitamin E is important for immune system function. In addition, these vitamins may also have additional therapeutic effects, such as inhibiting cancer, cardiovascular disease, and macular degeneration [101]. The incorporation of these oil-soluble vitamins into some foods and beverages is challenging because of their low water-solubility, chemical instability, and low bioavailability [102]. These challenges can be overcome using colloidal delivery systems. The encapsulation of oil-soluble vitamins within colloidal particles (which have a hydrophobic interior and hydrophilic exterior) increases their water-dispersibility. Moreover, encapsulation can increase their chemical stability by isolating them from the external environment and/or by including preservatives like antioxidants inside the particles [95]. Indeed, many plant proteins naturally exhibit antioxidant activity and can therefore be used to protect oil-soluble vitamins from degradation when used in formulated delivery systems. Studies have shown that nanoemulsions can be used to improve the water dispersibility and chemical stability of several oil-soluble vitamins [88]. Moreover, recent studies have shown that this type of system can be created entirely from plant-based ingredients [93].

Colloidal delivery systems can also be used to alter the gastrointestinal fate of oil-soluble vitamins, such as their chemical stability and bioaccessibility [103]. The effectiveness of these delivery systems depends on their composition and structure. For instance, the bioaccessibility of oil-soluble vitamins (A, D, and E) has been shown to increase with decreasing droplet size in nanoemulsions, which is attributed to the faster release and solubilization of the vitamins when lipid digestion occurs more rapidly [93]. Other critical factors for vitamin bioaccessibility are oil phase composition, interfacial properties, and additives [103]. In particular, the lipid phase used should form mixed micelles that are large enough to solubilize the vitamins released from the colloidal particles during digestion.

Colloidal delivery systems can also be designed to control the release pattern of the vitamins, which may be useful for extended-release applications. This approach can be used to reduce the potential toxicity effects that may occur if blood levels of vitamins are too high. This kind of delivery system can be prepared by modification of particle size, pore size, or interfacial layers. For instance, controlled release of vitamin D was achieved using zein nanoparticles by coating them with a layer of carboxymethyl chitosan [47].

### 5.2. Omega-3 Oils

Plant-based oils rich in omega-3 fatty acids, such as flaxseed and algal oils, may have health benefits, e.g., reducing inflammatory, cardiovascular, visual, and neurological diseases [4]. Eicosapentaenoic acid (EPA) and docosahexaenoic acid (DHA) are the two more potent forms of omega-3 fatty acids for human health. These two fatty acids can be obtained from fish or from algae (which are not really plants but are often used to formulate plant-based products). Alpha-linolenic acid (ALA) and stearidonic acid (SDA) are omega-3 fatty acids that can be directly obtained from plants, but they only undergo limited conversion to EPA or DHA inside the human body, which reduces their biological activity [4]. Some of the challenges associated with incorporating omega-3 fatty acids into functional foods and beverages are their low water-solubility, poor chemical instability, and low bioavailability. Again, these challenges can be overcome using colloidal delivery systems.

The water-dispersibility of omega-3 oils can be improved by trapping them inside colloidal particles that have hydrophobic interiors and hydrophilic exteriors, like nanoemulsions, SLNs, or NLCs. The tendency for omega-3 oils to oxidize can be reduced using several approaches. First, it is critical to avoid conditions that could promote oxidation when preparing omega-3 oil-loaded delivery systems, such as oxygen, light, heat, and transition metals. Second, the delivery systems themselves can be designed to inhibit oxidation, i.e., by adding antioxidants or chelating agents [104], by engineering the interfacial layers [105], or by encapsulating within microgels [54,106]. For instance, plant proteins such as those from lentils, peas, and fava beans, are natural antioxidants, which means they can be used to improve the oxidative stability of encapsulated ω-3 oils [66]. Interfacial engineering has been used to improve the oxidative stability of algae oil nanoemulsions by forming pea protein-flaxseed gum coatings around the oil droplets [94]. Encapsulation of ω-3 oil droplets inside alginate microgels has also been shown to improve their oxidative stability [54,106]. The bioavailability of omega-3 oils can also be increased by encapsulating them within colloidal delivery systems. For instance, the encapsulation of these oils within nanoemulsions has been shown to increase their bioaccessibility and bioavailability using in vitro and in vivo studies [107,108], which was mainly attributed to the faster and more extensive digestion of the lipids when they were present within small droplets.

### 5.3. Nutraceuticals

Nutraceuticals are substances found in foods that are not essential for human health and wellbeing but may promote human health if ingested regularly at sufficiently high levels. For instance, they may help to inhibit the development of heart disease, cancer, diabetes, or stroke, or they may improve mood, focus, or energy levels.

#### 5.3.1. Carotenoids

Carotenoids are naturally found in many fruits and vegetables, where they act as natural pigments and antioxidants [29,30]. Tomatoes and watermelons contain relatively high amounts of lycopene; carrots and spinach are rich in β-carotene; red peppers, papayas, and kale are good sources of xanthophylls. Some carotenoids exhibit vitamin A activity, whereas most of them exhibit other beneficial biological activities or therapeutic effects, including antioxidant activity or the ability to inhibit cardiovascular diseases or vision loss [109]. Carotenoids can also be isolated from their natural source and used as functional ingredients in foods. However, the bioavailability of carotenoids in natural sources and in fortified foods is often relatively low. Moreover, carotenoids have a very low water solubility and are chemically unstable, which also reduces their utilization and efficacy as nutraceutical ingredients [110]. Well-designed delivery systems can be used to enhance the bioactivity of carotenoids by increasing their water solubility, stability, and bioavailability.

Nanoemulsions are commonly used as delivery systems for carotenoids because of their versatility and ease of production. Like oil-soluble vitamins, the bioavailability of carotenoids depends on oil phase-type, oil concentration, droplet size, emulsifier type, additives, and various other factors. Typically, the bioaccessibility of β-carotene increases as the droplet size decreases because faster lipid digestion leads to faster carotenoid release and solubility [96,111]. The chemical stability of β-carotene in plant-based nanoemulsions has been enhanced by adding a plant-derived natural antioxidant (tannic acid) without adversely affecting the bioaccessibility [95].

Nanoemulsions can also be used as excipients to increase the bioaccessibility of carotenoids in fruits and vegetables. For instance, it has been shown that mixing carrots with excipient nanoemulsions greatly increased carotenoid bioaccessibility [112]. Another study showed similar results when mangoes were mixed with excipient nanoemulsions [97]. The nanoemulsions are rapidly digested in the small intestine, which leads to the formation of mixed micelles capable of solubilizing the carotenoids released from the fruits and vegetables, thereby increasing their bioaccessibility.

#### 5.3.2. Curcuminoids

Turmeric naturally contains a group of curcuminoids, such as curcumin, demethoxycurcumin, and bis-demethoxycurcumin, which are natural pigments that also exhibit several biological activities, such as antioxidant, anti-inflammatory, and anticancer activities [113]. Like many other nutraceuticals, curcumin is a hydrophobic and chemically labile substance that has low water solubility, poor stability, and low bioavailability. For this reason, many researchers have developed colloidal delivery systems to overcome these problems.

Nanoemulsions have been shown to be particularly effective at improving the functionality of curcumin [56]. For instance, they have been used to improve its water dispersibility, chemical stability, and bioavailability [51]. Nanoliposomes and biopolymer nanoparticles have also been shown to be effective for this purpose [51]. Curcumin can be incorporated into nanoemulsions by dissolving it within the oil phase prior to homogenization or by adding it after homogenization, e.g., using temperature-, solvent- or pH-driven methods [98]. Curcumin has a relatively small molecular weight (368 Da), which means that its bioaccessibility can still be relatively high when delivered in nanoemulsions prepared from short or medium-chain triglycerides (rather than long-chain ones) [114]. In contrast, carotenoids are too large to fit into the mixed micelles formed from the digestion products of these shorter triglycerides, and so long-chain triglycerides should be used to ensure good bioaccessibility.

#### 5.3.3. Polyphenols

Polyphenols are a diverse group of molecules found in plants, including flavonoids (e.g., anthocyanins, flavanols, flavanones, flavonols, flavanones, and isoflavones) and non-flavonoids (e.g., phenolic acids, xanthones, stilbenes, lignans, and tannins) [115]. Curcumin is actually a polyphenol that can be categorized as phenolic acid. Other well-studied polyphenols are quercetin, myricetin, epigallocatechin-3-gallate (EGCG), catechin, and anthocyanins from the flavonoid group, and tannic acid, gallic acid, and resveratrol from the non-flavonoid group. Polyphenols can exhibit strong antioxidant activities, which can help combat oxidative stress within the human body, as well as exhibiting several other possible health benefits, such as against cardiovascular disease, hypertension, diabetes, and cancer [32,115].

The water-solubility of polyphenols depends on their molecular structure—some are water-soluble, and some are oil-soluble. For instance, EGCG, anthocyanins, and phenolic acids are water-soluble, whereas curcumin, quercetin, and resveratrol are oil-soluble. Therefore, different strategies are required to encapsulate these components [116]. Water-soluble polyphenols may need to be encapsulated within colloidal particles with hydrophilic interiors to protect them from degradation during storage. For instance, anthocyanins have been encapsulated within the hydrophilic interiors of W/O/W emulsions that were formulated using polyglycerol polyricinoleate (PGPR) as an oil-soluble emulsifier and quillaja saponin as a water-soluble emulsifier [117]. Similarly, EGCG has been encapsulated in biopolymer microgels to protect it from degradation during storage and passage through the gastrointestinal tract, which led to an increase in its bioaccessibility [92].

Hydrophobic polyphenols need to be encapsulated in delivery systems containing colloidal particles with hydrophobic interiors and hydrophilic exteriors, like nanoliposomes, nanoemulsions, SLNs, NLCs, or some biopolymer nanoparticles. As an example, the water dispersibility, chemical stability, and bioavailability of hydrophobic polyphenols have been improved by encapsulating them within nanoliposomes, nanoemulsions, and zein nanoparticles [116]. Plant-based nanoemulsions have also been used to increase the stability and bioaccessibility of curcumin, resveratrol, and quercetin [100]. It should be noted that polyphenols can also be added to colloidal delivery systems as antioxidants to protect other nutraceuticals [118].

## 6. Potential Toxicity

The potential toxicology of colloidal particles should also be considered when designing delivery systems for bioactive agents. For instance, a burst release of a high concentration of vitamins or nutraceuticals might lead to a relatively high concentration of these bioactive agents in the bloodstream, which could have potentially harmful effects for some individuals. For instance, studies have shown that consuming high levels of vitamin E or carotenoids can promote cancer in smokers. Consequently, colloidal delivery systems that increase the bioavailability of these bioactive agents could cause problems in this population. Most kinds of colloidal particles are comprised of digestible components, such as lipids, proteins, or starches, and would be expected to be fully digested in the human gut, albeit at a faster rate than normal due to their high surface areas. Moreover, many colloidal particles made of indigestible components, such as dietary fibers, may disassemble as they move through the human gut. As a result, they would be expected to have similar biological impacts as conventional foods. However, small colloidal particles that were not digested and that remained intact could be absorbed by the body, which could have unknown effects. The potential of the biopolymer nanoparticles to promote oxidative stress and an immune response should also be considered [51]. In addition, some plant proteins cause allergic reactions in certain people, such as soy proteins. Thus, as new kinds of plant proteins are developed and utilized in foods, it will be important to establish their potential allergenicity. Additional information about the potential toxicology of edible colloidal particles can be found elsewhere [119].

## 7. Conclusions and Future Studies

One of the fastest-growing segments of the modern food industry is the plant-based foods market. Meat, fish, egg, and dairy analogs are being developed to address consumer concerns about the negative environmental, health, and animal welfare impacts of traditional animal-based foods. However, plant-based foods often do not have the same nutritional profiles as animal-based ones and may therefore benefit from being fortified with micronutrients or nutraceuticals. In this article, we have shown that colloidal delivery systems that are suitable for the encapsulation, protection, and delivery of vitamins and nutraceuticals can be assembled entirely from plant-based ingredients. Several types of delivery systems are available for this purpose, and the most appropriate one must be selected for a particular application. Moreover, the composition and structure should be controlled to enhance the water dispersibility, stability, and bioavailability of the encapsulated bioactive agents. These plant-based delivery systems may be particularly useful for the development of the next generation of healthier plant-based food products. For instance, they could be used to incorporate vitamins and nutraceuticals into these products, such as vitamins A, B, C, D, E, carotenoids, curcuminoids, and polyphenols, as well as natural pigments, flavors, and preservatives.

## Figures and Tables

**Table 1:** Encapsulation properties, stability, and gastrointestinal fate of different types of delivery systems for hydrophobic bioactives.

Delivery System Type	Size	Charge	Composition	Loading Properties	Stability	Digestion Properties	References
Nanoparticle, nanoliposome, nanoemulsion	Nanoparticle and nanoliposome: 99 nm; nanoemulsion: 168 nm	Nanoparticle: 20 mV; nanoliposome: −5.2 mV; nanoemulsion: −6.5 mV	Curcumin; zein nanoparticle; phospholipid nanoliposome; Tween 20 stabilized corn oil nanoemulsion	Loading capacity: nanoparticle: 12%; nanoliposome: 3%; nanoemulsion 0.4%	Chemical stability of curcumin in GIT: nanoemulsion ≈ nanoparticle > nanoliposome	Curcumin bioaccessibility: nanoemulsion (92%) > nanoliposome (74%) > nanoparticle (52%)	[51]
Nanoparticle	86–200 nm	−27 to −11 mV as increasing calcium level	Vitamin D; zein nanoparticles with different levels of carboxymethyl chitosan and calcium	Loading capacity decreased from 3.9 to 1.7%; encapsulation efficiency increased from 52% to 88% as increasing chitosan or calcium level.	Photochemical stability increased from ~70% to 80% with the chitosan coating, compared to 30% of free vitamin D.	Vitamin D release in the GIT decreased with the chitosan coating (~55%) compared to ~100% for zein nanoparticles.	[47]
Nanoparticle	267–728 nm as increasing calcium level	−23 to −14 mV as increasing calcium level	Resveratrol; zein-propylene glycol alginate-tea saponin complex at different calcium level	Encapsulation efficiency increased with tea saponin (58% to 77%), irrespective of calcium level. Loading capacity was the same for all treatments.	Tea saponin increased photo and thermal stability of resveratrol; calcium increased the environmental stability (thermal, pH, ionic) of the particle.	Calcium reduced the release of resveratrol in the stomach but promoted the release in the small intestinal phase.	[49]
Solid lipid nanoparticle (SLN), nanoemulsion	Corn oil: 0.5 μm; cocoa butter: 0.9 μm	Corn oil: −29 mV; cocoa butter: −42 mV	β-Carotene; cocoa butter, cocoa oil, corn oil, Tween 80	-	β-Carotene stability decreased in cocoa oil with a compact crystalized lipid core and in small oil droplet size.	Triglycerides (TAG) disappearance: 90% for both; bioaccessibility: 64% for corn oil, 82% for cocona butter.	[87]
Nanostructured lipid carrier (NLC)	94 nm	−24 mV	Astaxanthin; oil phase: glyceryl behenate, oleic acid, lecithin, α-tocopherol; aqueous phase: Tween 80 and EDTA	-	Carbonation and thermal pasteurization increased particle size and astaxanthin loss.	-	[85]
SLN, NLC	<200 nm	−40 to −20 mV	Curcumin; oil phase: glyceryl behenate (SLN) or mixed with oleic acid oil (NLC); aqueous phase: casein, Tween 80, pectin	Tween 80, oil blending, and pectin coating improved loading capacity and encapsulation efficiency (~35% to 66%).	The size of SLN was more stable than NLC during storage.	SLN showed less curcumin release than NLC.	[86]
Microgel	Alginate: 626 μm; carrageenan: 849 μm; mixture: 763 μm	Alginate: −24 mV; carrageenan: −16 mV; mixture: −21 mV	Corn oil droplets stabilized by Tween 80; gelling structure: alginate, carrageenan, and mixture	-	Alginate beads were intact, while carrageenan beads were deformed and dissociated in the GIT.	The free fatty acid (FFA) release rate decreased as carrageenan (~80%), mixture, alginate (~50%).	[57]
Emulsion, microgel bead	Emulsion: 0.27 μm; alginate bead: ~400 μm; chitosan bead: ~250 μm	Emulsion: −20 mV; alginate beads: −17 mV; chitosan beads: −23 mV at pH 7	Curcumin; emulsion: corn oil droplets stabilized by Tween 80; microgel beads: alginate vs. chitosan	High encapsulation of the lipid droplets in both alginate and chitosan beads.	At pH 7, curcumin degradation under 55 °C increased as: chitosan < emulsion < solution < alginate; at pH 3, the order was: emulsion < solution < chitosan < alginate.	-	[56]
Nanoemulsion vs. microgel	Nanoemulsion: 0.2 μm; microgel: ~500 μm	Nanoemulsion: −74 mV; microgel: −46 mV	Flaxseed oil nanoemulsion stabilized by quillaja saponin; alginate microgel bead; casein as an antioxidant	-	Particle size was the same under 55 °C. Microgel inhibited lipid oxidation (hydroperoxides and TBARS).	FFA release: ~100% for emulsion, ~50% for microgel	[54,91]
Microgel	Chitosan microgel was much smaller than gelatin microgel	-	(-)-Epigallocatechin gallate (EGCG); microgel beads: gelatin vs chitosan	Encapsulation efficiency: 95% for gelatin; 82% for chitosan	The initial release of the EGCG was faster in the chitosan microgel, but the final release was the same.	Release of EGCG reduced as: Gelatin > chitosan, which was significantly less than free EGCG.	[92]
Emulsion	0.15, 1.6, 11 μm	−68 to −57 mV	Vitamin A, D, E; soy oil emulsion stabilized by quillaja saponin	-	Oil droplet size remained unchanged during GIT. Vitamin stability in GIT was the same for different oil droplet sizes.	FFA release decreased from 125% to 99% as increasing oil droplet size. Bioaccessibility: vitamin A: 87%, 68%, 39%; vitamin D: 76%, 76%, 44%; vitamin E: 77%, 40%, 21% as increasing oil droplet size.	[93]
Emulsion	Pea protein: 36 μm; complex with flaxseed gum: 20 to 54 μm	Pea protein: 33 mV; complex with flaxseed gum: −3 mV	Algae oil emulsion stabilized by pea protein with or without flaxseed gum	-	The emulsion was stable in pure protein or high flaxseed gum level but unstable at low flaxseed gum level (0.01%). Flaxseed gum reduces lipid oxidation and the release of undesirable volatiles.	-	[94]
Emulsion	0.3–0.4 μm	−25 to −20 mV	Fish oil droplets stabilized by lentil, pea, and faba bean proteins	-	All emulsions were unstable in stomach or under different pH, ionic strength, temperature. Lentil protein emulsion was most stable. All plant proteins inhibited lipid oxidation.	FFA reached 100% for all samples.	[66,95,96]
Emulsion	Quillaja saponin: 0.13 μm; gum arabic: 0.33 μm	Quillaja saponin: −63 mV; gum arabic: −32 mV	β-Carotene; flaxseed oil droplets stabilized by either quillaja saponin or gum arabic, tannic acid as an antioxidant	-	Emulsions were relatively stable at 55 °C and in GIT.	Quillaja saponin slightly reduced lipid digestion to 90%. The β-carotene bioaccessibility was the same (45%) for all emulsions.	[95]
Emulsion	Tween 20, quillaja saponin and casein: 0.17 μm; lysolecithin: 0.33 μm; gum Arabic: 0.48 μm	Casein, lysolecithin and gum Arabic: −42 to −33 mV; quillaja saponin: −72 mV; Tween 20: −18 mV	β-Carotene, corn oil droplets stabilized by Tween 20, quillaja saponin, casein, gum arabic, or lysolecithin	-	Casein and lysolecithin stabilized emulsions were unstable in GIT.	FFA release: Tween 20 and quillaja saponin (>100%) > gum arabic (99%) > soy lysolecithin and casein (93%); β-carotene bioaccessibility: Tween 20 (62%) > quillaja saponin (56%) > casein (55%) > gum arabic (51%) > lysolecithin (25%).	[96]
Excipient emulsion	0.2, 0.5, 10 μm	−72 to −42 mV as increasing oil droplet size	Carotenoids from carrots; whey protein stabilized corn oil emulsion	-	The emulsion was unstable in the stomach phase.	FFA release: over 100% for 0.2 and 0.5 μm, >90% for 10 μm and bulk oil; Bioaccessibility: α-carotene and β-carotene decreased as increasing oil droplet size (32% to 7%).	[80]
Excipient emulsion	0.7–0.9 μm	-	Carotenoids from carrot or tomato purees, sucrose ester, stabilized emulsion, olive (OO), soybean (SO), or linseed (LO) oils	-	All emulsions were unstable in the stomach phase.	TAG disappearance: 43–44% for OO, 42–45% for SO, 34–36% for LO. Bioaccessibility: β-carotene from carrots: 13% for OO, 8–10% for SO and LO; cis-lycopene from tomato 27% for OO, 15% for SO and LO.	[79]
Excipient emulsion	<0.2 μm	LCT: −23 mV; MCT: −14 mV	Carotenoids and phenolics from mangoes; Tween 20 stabilized emulsion: corn oil (LCT) vs. MCT	-	All emulsions were stable in GIT.	FFA release: MCT (>100%) > LCT (80%). Phenolics bioaccessibility: 80%, 100%, 80%; carotenoids bioaccessibility: 35%, 60%, 80%, for buffer solution, MCT, LCT emulsions respectively.	[97]
Emulsion	<0.2 μm	−45 mV	Curcumin loaded by pH driven method, heat-driven method, or conventional oil loading method; corn oil emulsion stabilized by quillaja saponin	Encapsulation efficiency: pH driven (93%) > heat-driven (76%) > conventional oil loading (56%)	Emulsions were stable in GIT.	FFA release (~80%), curcumin stability (76–92%) and bioaccessibility (74–79%) were similar.	[98]
Nanoemulsion	~0.2 μm	−46 to −40 mV	Vitamin E acetate, quillaja saponin stabilized emulsions: corn oil (LCT) or MCT	-	-	FFA release: MCT (> 100%) > LCT (~80%); vitamin E bioaccessibility: LCT (39%) >MCT (17%); vitamin E conversion: LCT (29%) > MCT (17%)	[81]
Emulsion and nanoemulsion	0.2, 20 μm	−60 to −50 mV	Vitamin D; corn oil emulsion stabilized by quillaja saponin	-	-	FFA release: 100% to 69%; vitamin D bioaccessibility: 1.8 μg/mL, 0.5 μg/mL; bioavailability: 22 ng/mL, 18 ng/mL as increasing oil droplet size	[99]
Emulsion	<0.2 μm	−70 to −60 mV	Curcumin, resveratrol, and quercetin; quillaja saponin stabilized emulsion: coconut, sunflower, and flaxseed oil	Encapsulation efficiency: 70–90%, higher in long-chain TAGs	Long-chain TAG promoted higher gastrointestinal stability of polyphenol.	Long chain TAG and resveratrol retarded lipid digestion. Resveratrol: 86%, 80%, 77%; curcumin: 52%, 53%, 59%; quercetin: 48%, 75%, 69%, for coconut oil, sunflower oil and flaxseed oil respectively.	[100]

## Data Availability

Not applicable.
